# A visualization tool for individual gene expression profiles among males and females in GTEx tissues

**DOI:** 10.1186/s13293-025-00796-3

**Published:** 2025-12-04

**Authors:** Kuo-Feng Tung, Wen-chang Lin

**Affiliations:** Institute of Biomedical Sciences, Academia Sinica, Taipei, 115 R.O.C Taiwan

**Keywords:** Protein-coding gene, Sexual dimorphism, GTEx project, Tissue specific expression, Bioinformatic visualization interfaces, Gini coefficient index

## Abstract

Sexual dimorphism has been implied to certain human physiology and diseases. This topic has recently garnered more attention, highlighting individual variances in precision medicine and individualized clinical trials. It is recognized that individual gene expression variations in males and females could have profound physiological impacts. Tissue specific expression profiles determine protein-coding gene activities and contribute additional physiological variations. Therefore, tissue specific gene expression profiles should be comprehensively analyzed among individual human subjects. In this report, we developed a user-friendly bioinformatic tool to visualize gene expression levels and variances across tissue samples, aiming to facilitate research into potential sexual dimorphism genes. The Gini coefficient metric was used with the most recent GTEx V10 datasets to examine variations in the expression profiles of human protein-coding genes across 43 tissue subtypes. Next, these variations were specifically evaluated using the Gini coefficient index for male and female individuals across all tissue subtypes. Our web-based visualization tool generated tissue specific expression profiles for individual male and female samples. It concurrently illustrates expression levels and variation comparisons between male and female groups across all tissue subtypes. Although most protein-coding genes had similar expression variation patterns between the two sexes, several genes exhibited distinct variations for some tissue subtypes, as indicated by their significant Z-scores in Gini index disparities. Users can explore differentially expressed protein-coding genes across tissue subtypes or search for genes of interest in the Tissue Prominent Sexual Dimorphism Gene database (https://tpsdg.ibms.sinica.edu.tw). This database can be employed to visualize expression levels and variations among individual samples within specific tissues, thereby facilitating future research into divergently expressed protein-coding genes in the human population.

## Highlights

We developed a freely accessible Web-based tool for visualizing protein-coding gene expression in individual male and female tissue samples.

The Gini index was used to determine potential differences between male and female individuals.

The tool simultaneously depicts gene expression levels and variations between male and female groups across multiple tissue subtypes.

Our tool offers user-friendly query and selection functions for comparing expression profiles across GTEx tissue subtypes.

## Background

Historically, the majority of tissues in male and female individuals were assumed to be biologically and physiologically similar, with the exception of those in the reproductive system. However, in recent years, there has been an increasing emphasis on the significance of sexual dimorphism in clinical practices and biomedical researches [1]. Sex-related differences are commonly observed in the prevalences and outcomes of certain human diseases [2]. In addition, some clinical trials of prescription drugs have yielded inaccurate or inadequate data, leading to subsequent withdrawn of the drug from market, likely attribute to the dominant male subjects recruited in the original study designs [1]. Therefore, individual male or female patients may substantially differ in medication responses and treatment outcomes. Modern clinical trials and medical investigations have paid increasing attention to both sexual dimorphism and gender equity issues. Moreover, the concept of individual variation has been emphasized by the recent implementation of the N-of-1 clinical trial design in precision medicine, which facilitates therapeutic evaluation at the individual level and acknowledges interpatient variability [3]. Latest studies have reported new intriguing findings related to individual variations in sexual dimorphism [4, 5].

Although female individuals are assumed to have relatively high physiological variability because of the menstrual cycle, emerging evidence suggests this assumption should be revised. Research involving big data and meta-analysis of multiple physiological traits have revealed a greater variation in energy expenditure in males than in females [6]. Studies have also indicated that the menstrual cycle is not associated with significant variations in female individuals [4, 6]. These findings are supported by large scale animal experiments, which revealed that male mice presented higher variability in certain behavioral and morphological traits than do female mice [7–9]. Sexual dimorphism studies have examined sex-biased genes at the molecular level to detect difference using average gene expression between male and female cohorts [10]. In addition, many studies have also reported tissue specific profiles for differentially expressed sex-biased genes [11–16]. In these reports, it has been noted that female individuals exhibit greater variability in immunity-related genes than do male individuals [9, 17].

In this study, we used the Gini coefficient index to explore differences in the expression patterns of protein-coding genes between male and female individuals. This index, introduced by Dr. Corrado Gini, measures economic income inequality. It is calculated as the ratio of the area between the line of perfect equality and the Lorenz curve to the total area under the line of perfect equality [18]. When all measurements are identical, the minimum value is 0, whereas the theoretical maximum of the inequality is 1. Its calculation offers several advantages, including direct comparisons of variance levels across different groups and the ability to handle undetectable expression values for certain protein-coding genes [19]. Recently, the Gini index has been used to assess sample distribution inequality across various domains, including differences in gene expression [19, 20]. This index can reliably identify stable expression patterns in human housekeeping genes, particularly with a large number of samples [21]. Most studies have investigated difference in average gene expression levels between male and female individuals. However, very few studies have examined individual variations in tissue related gene expression patterns. Therefore, individual male and female variations should be more emphasized in studies exploring human variation and sexual dimorphism, and gene expression should not be assumed to be uniform across individual human samples. It might be necessary to depict specific tissue expression profiles in individual samples to reflect variations in gene expression [10, 12, 22]. Therefore, a new bioinformatic tool is required to analyze individual-level variations in gene expression across tissue subtypes.

The GTEx project is the optimal resource for such multiple tissue transcriptomic expression analysis involving a large number of human subjects [23]. This prominent project provides the gene expression profiles of non-cancerous normal tissues across numerous individual human donors [14, 15, 23]. By employing nearly 20,000 human tissue samples from the most recent GTEx V10 dataset, one can considerably investigate the tissue expression patterns of potential sex-biased protein-coding genes. In this report, we used this dataset to establish a novel tool that can easily visualize protein-coding gene expression patterns and variations in different tissues among male and female individual samples. Several important databases have been developed to study sexual dimorphism genes [24–26]. For example, voyAGEr is a web-based database that facilitates the exploration of age-related gene expression patterns and provides information on expression differences between male and female individuals [24]. SexAnnoDB is a database designed to study sex-based differences in cancer related genes and drug molecules [26]. Despite the benefits of these databases, few dedicated bioinformatic tools have been developed to visualize gene expression profiles across different tissue subtypes, especially with individual variations. Therefore, to facilitate the interrogations on variable expression genes in human individuals, we would like to present this user-friendly bioinformatic tool that can easily visualize protein-coding gene tissue specific expression patterns and variations across male and female individual samples. Particularly, this tool enables users to simultaneously interrogate the Gini index profiles (variations) and expression levels of protein-coding genes across various tissue subtypes.

## Construction and content

### Gene expression datasets of GTEx tissues

The GTEx V10 dataset was released in late 2024, which contains 12% more RNA-seq samples than does the previous V8 version. The gene expression dataset (gene TPM level) utilized in this study was directly obtained from the GTEx website (https://www.gtexportal.org/home/downloads/adult-gtex/overview). All retrieved data contains no participant data and adheres to the NIH Genomic Data Sharing guideline. This dataset contains 54 gene expression files that provide RNA-seq TPM data on distinct tissues and cell lines, amounting to 19,788 samples from 946 donors. To specifically analyze sex-biased gene expression, we used only normal tissue expression information and thus excluded two cell line datasets (cells_clultured_fibroblasts and cells_ebv-transformed_lymphocytes). Subsequently, we removed kidney_medulla data (with only 11 samples) and data on eight other reproductive system tissues involving only single-sex information (cervix-ectocervix, cervix-endocervix, fallopian tube, ovary, prostate, testis, uterus, and vagina). The remaining 43 tissue subtypes were used for further Gini index calculation of male and female donor samples. Table 1 lists the number of tissue samples, which range from several dozen to a few hundred in respective tissues. In addition, the GTEx_Analysis_v10_Annotations_SubjectPhenotypesDS file was utilized to extract sample attribute data. Finally, the GTEx sample ID was used to extract the age and sex features of each sample in respective tissue subtypes.

**Table 1:** Numbers of male and female donor samples and protein-coding genes with significant divergent expression in 43 GTEx tissue subtypes

GTEx tissue subtypes	Male samples	Female samples	Male variable genes (M > F)	Female variable genes (M < F)
adipose_subcutaneous	481	233	49	7
adipose_visceral_omentum	407	180	5	1
adrenal_gland	184	111	27	8
artery_aorta	308	164	25	33
artery_coronary	168	100	64	8
artery_tibial	471	220	23	9
bladder	48	29	2	5
brain_amygdala	129	52	1	0
brain_anterior_cingulate_cortex	177	56	18	1
brain_caudate_basal_ganglia	224	76	7	4
brain_cerebellar_hemisphere	202	75	10	16
brain_cerebellum	194	72	19	29
brain_cortex	195	75	2	14
brain_frontal_cortex_ba9	201	68	0	29
brain_hippocampus	190	65	2	2
brain_hypothalamus	193	64	2	3
brain_nucleus_accumbens_basal ganglia	212	73	2	1
brain_putamen_basal_ganglia	193	61	5	23
brain_spinal_cord_cervical	136	68	4	20
brain_substantia_nigra	133	50	14	49
breast_mammary_tissue	336	178	281	18
colon_sigmoid	268	151	8	4
colon_transverse	307	172	14	21
esophagus_gastroesophageal_junction	266	137	34	24
esophagus_mucosa	399	215	3	18
esophagus_muscularis	368	193	35	25
heart_atrial_appendage	318	143	2	2
heart_left_ventricle	308	144	9	8
kidney_cortex	80	24	1	4
liver	184	78	1	13
lung	414	190	13	8
minor_salivary_gland	129	52	21	15
muscle_skeletal	552	266	3	28
nerve_tibial	459	211	57	15
pancreas	223	139	21	11
pituitary	229	84	29	18
skin_not_sun_exposed_suprapubic	445	206	29	8
skin_sun_exposed_lower_leg	506	248	8	3
small_intestine_terminal_ileum	135	72	1	6
spleen	176	101	53	1
stomach	259	148	13	13
thyroid	459	225	11	13
whole_blood	541	262	7	22

The GTEx datasets were retrieved from GTEx website, and Gini index values were determined for protein-coding genes in male or female cohorts as described. The number of autosomal protein-coding genes with significant sex-biased expression variations (M > F or M < F) was determined using the default settings in TPSDG database (a Z-score value of ≥ 2.58 and a TPM value cutoff of 1.0)

### Protein-coding gene annotations from GENCODE and MANE datasets

GENCODE version 39 was applied to annotate human protein-coding genes in the GTEx V10 analysis pipeline, therefore, we retrieved this GENCODE annotation file for showing protein-coding gene information. The gene type feature was used to obtain protein-coding genes in the retrieved GTEx main expression datasets. We then used MANE gene list as the standard protein-coding reference. The MANE project is a jointly developed effort by the National Center for Biotechnology Information (NCBI) and European Bioinformatics Institute (EBI) to provide unified standard reference annotations for human protein-coding genes [27]. In this study, the most recent annotation list (MANE.GRCh38.v1.4.summary.txt) was retrieved from the NCBI website. Then, MANE-select transcript information was extracted using the MANE_status feature column and also other annotation features such as gene symbol, gene name, chromosome location, Ensembl gene ID, Ensembl transcript ID, Ensembl protein ID, NCBI gene ID, NCBI RefSeq NM ID, and RefSeq NP ID. Although version 1.4 of the MANE dataset contains 19,338 gene records, besides annotation differences, it is recognized that not all genes expressed in respective tissue subtypes.

### Expression variations analyses using the Gini index

In previous study, we used Gini coefficient index for assessing the protein-coding gene expression variations [21]. Python and NumPy were used to analyze differences in gene expression, evaluated in terms of expression averages, coefficients of variation, and the Gini index. Herein, the Gini index values for male as well as female tissue samples were calculated to examine individual gene expression variations across 43 tissue subtypes.

We further applied Z-score (standard score) to identify potential extreme expression outliers. The Z-score is calculated as follows: (raw data value - average value)/standard deviation. While we would like to visualize all male and female samples in each tissue subtype in order to examine all variations in respective individuals. However, we noted certain some extreme outliers, presumably resulting from NGS technical problems, that could affect the overall expression levels and Gini index values. Therefore, we tried to include the majority of expression variations among individual samples. We decided to exclude the singleton sample with a gene expression Z-score value more than 15 within each tissue subtype and a mean Gini index value is above 0.97. Subsequently, the gene expression levels and Gini index values were then recalculated after removing these extreme outlier sample values.

### Construction of the tissue prominent sexual dimorphism gene database

The Tissue Prominent Sexual Dimorphism Gene (TPSDG) database is hosted in a web-hosting Docker engine running on an Ubuntu Linux server. This TPSDG database is implemented in PHP programming language, hosted on an Apache web server, and integrated with a MySQL database. A JavaScript D3 package is also implemented in the webpage for interactive graphical display of transcript expression levels [28]. All GTEx sample expression data for protein-coding genes from the GTEx V10 datasets are stored in the MySQL database for the TPSDG web interface. In the present study, we focused on generating useful visualization charts for interrogations on the expression levels and variations for protein-coding genes in male and female groups across 43 tissue subtypes. Some of the data statistical analysis and graph illustration figures were performed using the GraphPad Prism (version 10; GraphPad Software, Boston, MA, USA). All gene expression data in the TPSDG database are freely accessible at https://tpsdg.ibms.sinica.edu.tw.

## Utility and discussion

### Brief summary of the GTEx V10 data

In this study, we developed a web-based bioinformatic tool to visualize the protein-coding gene expression profiles of male and female individual samples across 43 tissue subtypes. It should be emphasized that the GTEx project provides multiple gene expression information in human “normal tissue” samples, and we used the most recent V10 datasets to develop this bioinformatic webtool for examining the expression variations in protein-coding genes among male and female individuals. This updated release version contains 12% more RNA-seq samples (19,788 human tissue samples) and 23% more samples for eQTL analyses. Although the GTEx project is the primary gene expression data resource for a large number of human tissue samples, this project is not established as a specific disease cohort. Therefore, direct discovery of disease-causing genes by using only our web-based tool remains limited. Nonetheless, the GTEx dataset is still the optimal resource for tissue specific patterns of protein-coding gene expression in a large number of male and female individuals. Our TPSDG web-based tool can reveal tissue specific variations in protein-coding gene expression, thereby elucidating interindividual gene expression patterns in males and females.

**Fig. 1:**
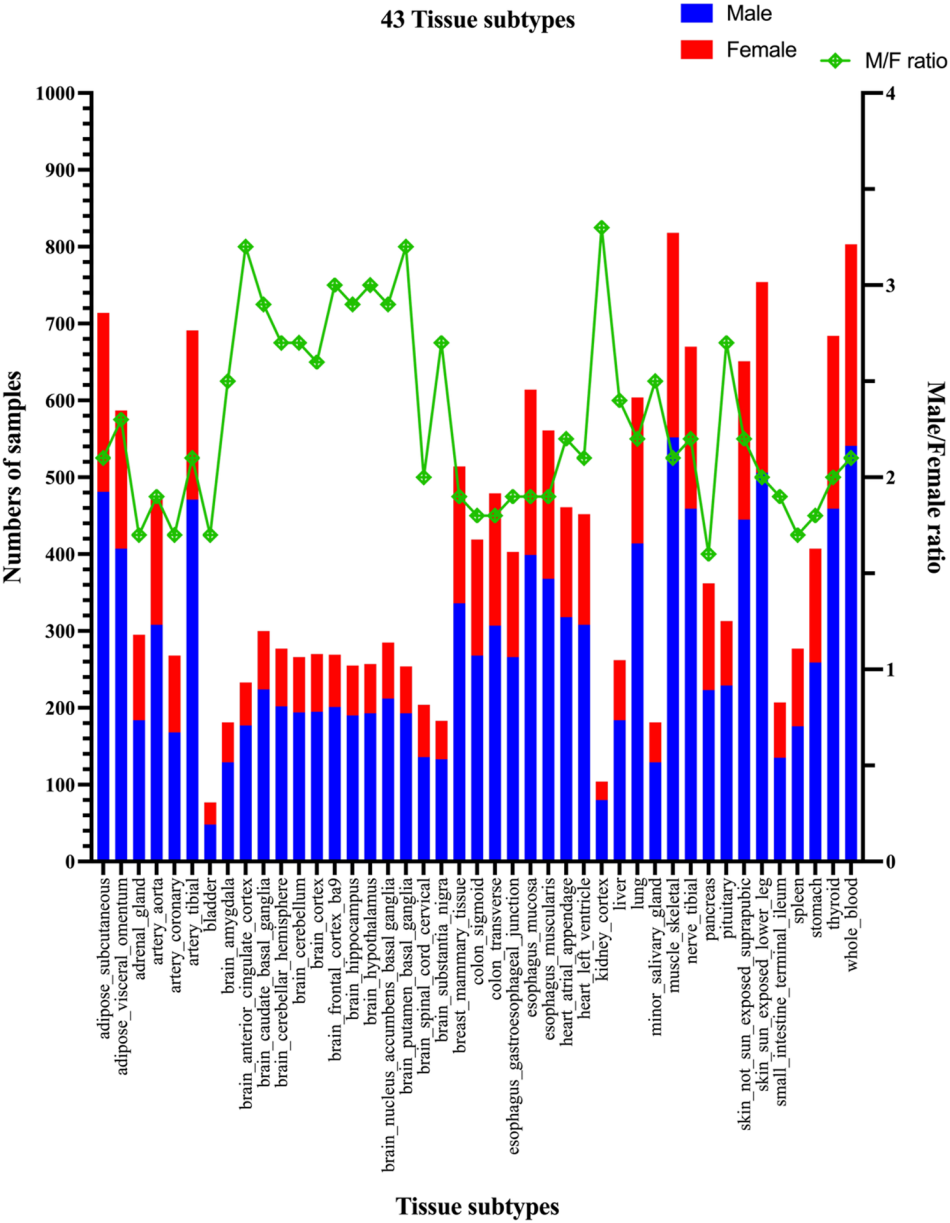
Illustration of numbers of samples and male to female sample ratio across the 43 GTEx tissue subtypes. The stacked bar graph (blue and red colors) displays the donor samples (male and female) for each tissue. The green square symbol indicates the male to female sample ratio. The number of male donor samples was higher than that of female donor samples in all GTEx tissue subtypes

After excluding 11 gene expression datasets involving either two cell lines, single-sex tissues, or small sample size, we have used remaining 43 tissue subtypes with both male and female samples from the GTEx project. A total of 17,338 samples were obtained in these 43 tissues: 11,807 male samples and 5,531 female samples (Fig. 1; Table 1). The number of samples ranged from several dozen to a few hundred in male or female groups in different tissue subtypes (Table 1). As shown in Fig. 1; Table 1, all tissue subtypes were dominated by male samples, with significant variations in the number of samples across different tissues. The ratio of male to female samples ranged from 1.6 (pancreas) to 3.3 (kidney_cortex), with an average of 2.3 across all tissues. As illustrated in Fig. 1, a total of 11 tissue subtypes were identified in the brain, the majority of which were associated with substantially high male to female ratios (except for brain_spinal_cord_cervical). Regarding the number of samples for each tissue subtype, as described previously [29], the most abundant tissues are muscle_keletal (818 samples); whole_blood (803 samples); skin_sun_exposed (754 samples); adipose_subcutaneous (714 samples) and artery_tibial (691 samples).

**Fig. 2:**
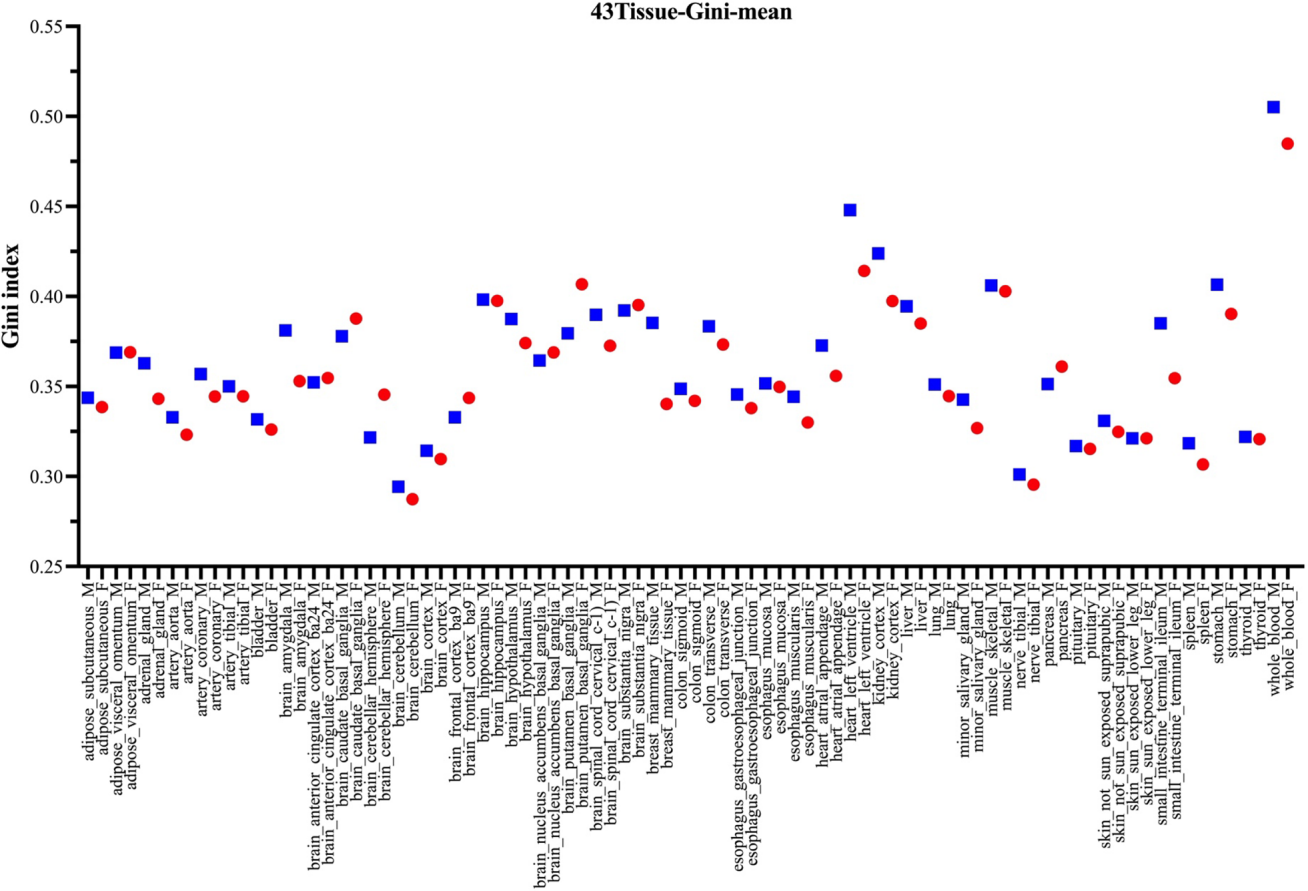
Distribution of Gini index values for males and females across the 43 GTEx tissue subtypes. The average Gini index values for protein-coding genes were calculated for each tissue subtype (blue squares denote male samples, whereas red circles denote female samples). Similar Gini index values were observed for male and female cohorts across the majority of tissue subtypes. Whole_blood exhibited the highest Gini index values, whereas brain_cerebellum exhibited the lowest Gini index

We utilized the Gini coefficient index for evaluating variations in protein-coding gene expression among the male individuals and female individuals. For each tissue subtype, we calculated the Gini index values of respective protein-coding genes in male group as well as in female group. As shown in Fig. 2, the Gini index values were similar for the majority of tissue subtypes, with a small difference noted between Gini-index-male and Gini-index-female groups. In male samples, the most diverse tissue types were whole_blood; heart_left_ventricle; kidney_cortex; stomach and muscle_skeletal. In female samples, the most diverse tissue types were whole_blood; heart_left_ventricle; brain_putamen_basal_ganglia; kidney_cortex and muscle_skeletal. In summary, blood emerged as the most diverse tissue subtype examined, suggesting a complicated individual immune status and hemopoietic cell composition, as noted by a previous report [17]. Among all tissues, Kidney_cortex might be less confident in this analysis, due to its relatively small number of samples (104 samples). Interestingly, the largest variation difference between Gini-index-male and Gini-index-female groups was observed for breast_mammary_tissue, consistent with the results of a prior publication [30].

### Main user interface illustrating tissue expressions and variations

**Fig. 3:**
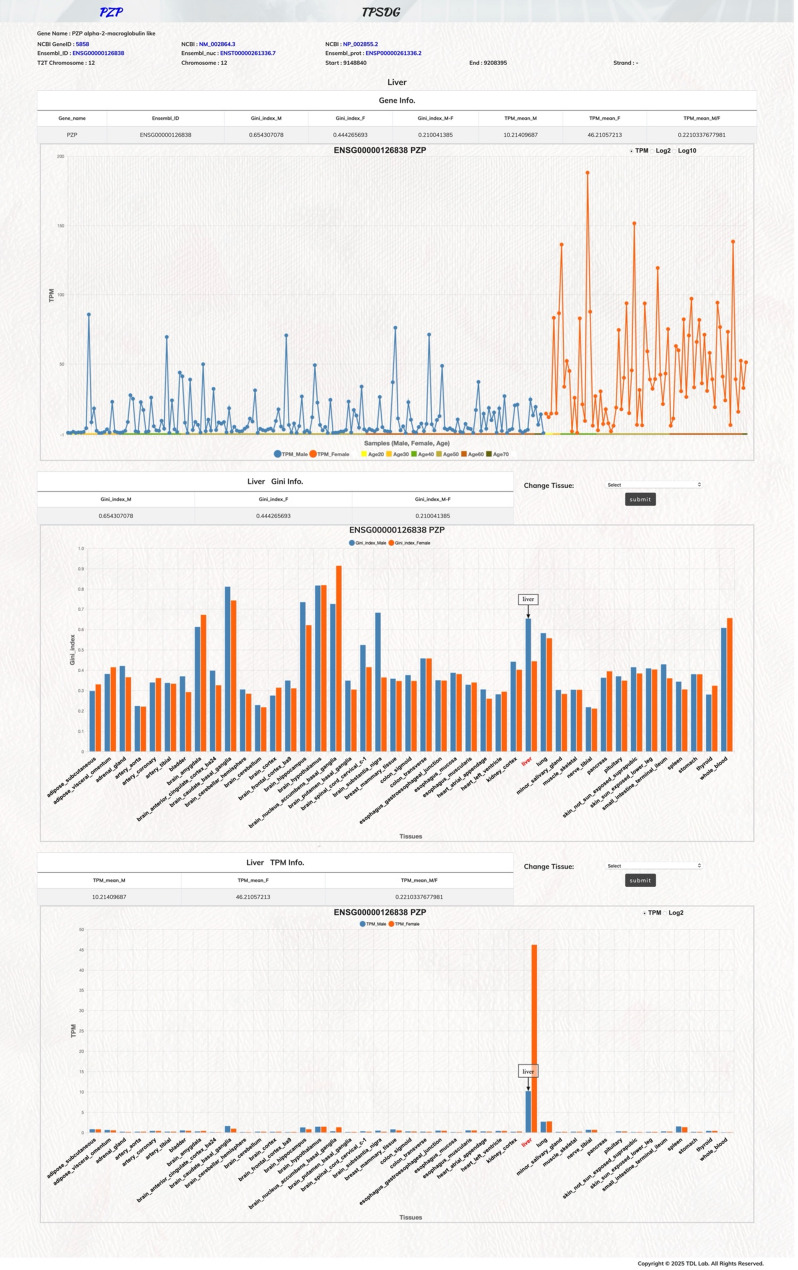
Webpage interface for PZP expression (liver tissue) in the TPSDG database. The graphical interface was developed to analyze specific protein-coding genes. The fundamental genetic information on PZP gene is presented in the upper panel. The expression of PZP gene in liver tissue in each sample is illustrated in the main graph (male: blue; female: orange). The middle and lower bar graph panels depict the distribution of Gini index and TPM values among male and female cohorts across all 43 tissue subtypes. After interrogating all data illustrated in these panels, users can switch the tissue subtype through a pull-down menu to further examine the individual sample expression profiles of PZP gene in another tissue subtype

In this novel Tissue Prominent Sexual Dimorphism Genes database (TPSDG), the main goal is to provide users a visualization webtool for exploring human protein-coding gene expression profiles among male and female individual samples for a particular tissue of interest (Fig. 3). In addition, it offers user-friendly illustration concurrently on the gene expression variations (Gini index) and gene expression levels (TPM) among male and female groups across all 43 GTEx tissue subtypes. Using the PZP gene as an example [15], the TPSDG gene information webpage displays essential basic protein-coding gene information at the top (Fig. 3), including Gene Name; NCBI Gene ID; NCBI NM ID; NCBI NP ID; Ensembl Gene ID; Ensembl Nuc ID; Ensembl prot ID and chromosome position information. The average gene expression information and Gini index value information for male and female groups are also listed here; along with the calculated TPM and Gini index differences between the two sexes. In the main graphic panel (Fig. 3), the PZP gene expression values of each individual sample are plotted for male (blue-color) and female (orange-color) donors. Within each sex group, individual samples are arranged by age groups (Age20; Age30; Age40; Age50; Age60; Age70 and above). Users may move the mouse cursor over a selected sample point to display detailed information on that tissue sample. The Y-axis scale may be modified to display TPM, Log2-TPM, or Log10-TPM for a clearer visualization of gene expression patterns.

As described, certain protein-coding genes exhibit considerable tissue specific variations in expression levels. To facilitate the comparison of tissue subtype expression and variation profiles, our TPSDG webtool is particularly designed to simultaneously display simple bar graphs for male and female groups across all 43 tissue subtypes (the middle and lower graphic panels). As shown in Fig. 3, PZP gene is a highly expressed protein-coding gene in liver tissue [15]. It is expressed at higher levels in female samples than in male samples; however, its expression diversity is greater in male samples than in female samples. Users can readily analyze expression and variation profiles across all 43 tissue subtypes on the integrated TPSDG webpage and switch to a different tissue subtype of interest by using the pull-down option. This unique functionality enables biomedical researchers to examine tissue specific individual expression variations of specified protein-coding genes.

### Initial TPSDG query web interface

This TPSDG tool was designed to analyze variations in gene expression among individual samples across certain tissue subtypes. Its intuitive query webpage enables users to select a tissue subtype at the beginning of the database query process. Alternatively, users can directly select a gene of interest by its gene symbol and then choose a tissue subtype to display relevant gene specific information, as illustrated in Fig. 3. Selecting a specific tissue subtype for analysis enables users to explore possible divergently expressed protein-coding genes, as determined by the difference in Gini index values, between male and female cohorts. In the present study, we observed that most protein-coding genes exhibit minor differences between male and female groups (Gini index difference within the range of *±* 0.1). To identify those protein-coding genes with large variations, we calculated the Gini index difference (depicted as *Gini index M-F* feature) as follows: Gini-index-male minus Gini-index-female. The Z-score of *Gini index M-F* is further applied for discovering genes with substantial Z-score differences between male and female groups. Users can then select genes of interest for further interrogations by first selecting Z-score cutoff (1.96 or 2.58). A Z-score of 1.96 corresponds to a probability of 5%; where a Z-score of 2.58 corresponds to a probability of 1% in sample distributions. Users can also choose the expression levels of protein-coding genes (overall TPM values: 0.1; 0.5; 1.0; 5 and 10); as well as autosome genes or sex chromosome genes (chromosome: 1–22 or XY).

One can initiate the investigation of divergently expressed genes by analyzing higher divergent genes in female individuals (M < F) or in male individuals (M >F) within each tissue subtype. Depending on the user-defined criteria, matched relevant protein-coding genes are listed on a table-oriented webpage. This webpage presents summarized variation and expression data (Gini_index_M; Gini_index_F; Gini_index_M-F; Gini_index_M-F_Zscore; TPM_mean; TPM_mean_M; TPM_mean_F; and TPM_mean_M/F). These data enable users to further select a gene of interest for a comprehensive visualization of its expression profile, as described in a previous section. On this table-oriented view page, all features can be sorted by selecting the column title icons. Using the default selection settings (a Z-score value of *≥* 2.58 and an expression TPM value cutoff of 1.0), we determined the numbers of genes whose expression different significantly between male and female cohorts (Table 1). Tissue specific differences were noted in human protein-coding genes, which highlights the utilities of this TPSDG webtool. The patterns of gene expression in breast_mammary_tissue were the most variable in male individuals. Recent studies have emphasized the importance of individual subject differences in human populations in addition to examining with averaged expression values [5]. Statistical analyses of individual variations revealed gene expression with sex-specific variability across tissues. These findings highlight the tissue specific expression characteristics of sexual dimorphism genes and the significance of examining tissue specific expression patterns.

Accordingly, our TPSDG webtool may serve as a practical and user-friendly resource for examining individual-level variations in gene expression across more than 40 tissue subtypes. For example, analysis of COMP gene (the gene that encodes Cartilage Oligomeric Matrix Protein), associated with Multiple Epiphyseal Dysplasia (MED), revealed elevated expression levels in male artery_ tibial and nerve_ tibial tissue samples. The prevalence of Multiple Epiphyseal Dysplasia is roughly three-fold higher in male individuals than in females [31]. Our visualization tool only illustrates divergent expression patterns for COMP gene in male samples; therefore, it alone is not suitable for further human disease implications. Besides two sexes, we also noticed that gene expression patterns differed between younger and older age groups with this visualization tool. For example, the expression level of HPR gene (the gene that encodes haptoglobin-related protein) was higher in the small intestine of younger male individuals than in that of older male individuals. This gene exhibits elevated expression in male patients with lymphoma in one report [32]. We further noted that the expression of STATH gene (the gene that encodes statherin) was higher in the pituitary gland of older male individuals than in that of younger male individuals. This statherin protein mediates the function of male sex accessory glands as reported [33]. It should be emphasized that this TPSDG database is limited in displaying only expression profiles for male and female individual samples in various tissue subtypes.

As implicated in eQTL studies, individual specific variations in gene expression could influence human physiology. There are certain technological challenges in library preparation and NGS sequencing mistakes. Therefore, we initially conducted limited data cleanup to exclude data records with extremely precarious expression levels (those record with Z-scores of over 15). However, after data clean-up, some individual samples still exhibited high expression levels for certain protein-coding genes. Subsequent analysis found unique single sample patterns, where elevated gene expression levels from the same donor sample ID appeared across several tissues. For example, two interferon-associated genes (IFI6 and IFIT3) exhibited significant higher expression profiles, particularly across several brain subtypes derived from a single GTEx-X4EP donor sample: brain_cerebellar_hemisphere; brain_cerebellum; brain_frontal_cortex; brain_putamen_basal_ganglia; brain_spinal_cord; brain_substantia_nigra. The involvement of various tissue subtypes suggests possible biological relevance, particularly relating to innate immune responses in this specific donor, rather than merely reflecting technical errors from library preparation and sequencing procedures. Accordingly, this user-friendly TPSDG database was designed to accommodate and display male and female samples with variable expression patterns across tissue subtypes.

The GTEx project serves as an invaluable resource of extensive transcriptome datasets derived from various human tissue types. However, it is not a particular designed as a disease-oriented cohort collection with specific aims and estimated patient sizes. Therefore, it should be utilized exclusively to analyze differences in gene expression variations between male and female individuals in the general human population. Notably, it may not be suitable for direct investigations of particular disease-related genes. Our TPSDG webtool attempted to reflect the diverse variations in tissue expression patterns among individuals. It can serve a unique resource for simultaneous analysis of protein-coding gene expression and individual variations across tissue subtypes. Additional biological experiments are required to gain comprehensive insights into these differentially expressed protein-coding genes.

## Conclusion

Sexual dimorphism has been linked to certain human diseases. Research into personal genomics has indicated that variations in individual human gene expression are crucial for understanding physiological distinctions and potential disease outcomes. In this report, we used the Gini coefficient and the newest GTEx V10 datasets to analyze the patterns of human protein-coding gene expression in nearly 20,000 tissue samples. We also examined variations of protein-coding gene expression across tissue subtypes for both male and female individuals. Our primary goal was to visualize the tissue specific gene expression patterns of individual samples across multiple tissue subtypes, since prior studies exploring sexual dimorphism genes have mostly utilized averaged expression values. While it is advocated for more focus on the sexual dimorphism, few dedicated resources are available for analyzing gene expression patterns in individual male and female samples. It is beneficial to examine potential sex-biased protein-coding genes exhibiting significant variable patterns in specific tissues between male and female cohorts. Although some limitations remain in direct investigations of human disease genes, the GTEx dataset remains to be the most comprehensive resource for protein-coding gene expression across a wide range of normal human tissues. Because of its user-friendly interface, our TPSDG database enables simultaneous analysis of gene expression levels and variation differences between male and female individuals across 43 GTEx tissue subtypes. Thus, this bioinformatic webtool may be useful for biological researchers investigating divergently expressed protein-coding genes across different tissues.

## Data Availability

All data examined in this research is included within this publication and can be accessed freely through the TPSDG weblink: [https://tpsdg.ibms.sinica.edu.tw]. All original gene expression datasets were obtained from the GTEx project website.
